# A domain knowledge enhanced yield based deep learning classifier identifies perineural invasion in oral cavity squamous cell carcinoma

**DOI:** 10.3389/fonc.2022.951560

**Published:** 2022-10-24

**Authors:** Li-Yu Lee, Cheng-Han Yang, Yu-Chieh Lin, Yu-Han Hsieh, Yung-An Chen, Margaret Dah-Tsyr Chang, Yen-Yin Lin, Chun-Ta Liao

**Affiliations:** ^1^ Department of Pathology, Chang Gung Memorial Hospital and Chang Gung University, Taoyuan, Taiwan; ^2^ Department of Strategic Technology, JelloX Biotech Inc., Hsinchu, Taiwan; ^3^ Department of Power Mechanical Engineering, National Tsing Hua University, Hsinchu, Taiwan; ^4^ Department of Otorhinolaryngology, Head and Neck Surgery, Chang Gung Memorial Hospital and Chang Gung University, Taoyuan, Taiwan

**Keywords:** oral cavity squamous cell carcinoma, perineural invasion, deep learning, artificial intelligence, digital pathology

## Abstract

**Background:**

Perineural invasion (PNI), a form of local invasion defined as the ability of cancer cells to invade in, around, and through nerves, has a negative prognostic impact in oral cavity squamous cell carcinoma (OCSCC). Unfortunately, the diagnosis of PNI suffers from a significant degree of intra- and interobserver variability. The aim of this pilot study was to develop a deep learning-based human-enhanced tool, termed domain knowledge enhanced yield (Domain-KEY) algorithm, for identifying PNI in digital slides.

**Methods:**

Hematoxylin and eosin (H&E)-stained whole-slide images (WSIs, n = 85) were obtained from 80 patients with OCSCC. The model structure consisted of two parts to simulate human decision-making skills in diagnostic pathology. To this aim, two semantic segmentation models were constructed (i.e., identification of nerve fibers followed by the diagnosis of PNI). The inferred results were subsequently subjected to post-processing of generated decision rules for diagnostic labeling. Ten H&E-stained WSIs not previously used in the study were read and labeled by the Domain-KEY algorithm. Thereafter, labeling correctness was visually inspected by two independent pathologists.

**Results:**

The Domain-KEY algorithm was found to outperform the ResnetV2_50 classifier for the detection of PNI (diagnostic accuracy: 89.01% and 61.94%, respectively). On analyzing WSIs, the algorithm achieved a mean diagnostic accuracy as high as 97.50% *versus* traditional pathology. The observed accuracy in a validation dataset of 25 WSIs obtained from seven patients with oropharyngeal (cancer of the tongue base, n = 1; tonsil cancer, n = 1; soft palate cancer, n = 1) and hypopharyngeal (cancer of posterior wall, n = 2; pyriform sinus cancer, n = 2) malignancies was 96%. Notably, the algorithm was successfully applied in the analysis of WSIs to shorten the time required to reach a diagnosis. The addition of the *hybrid intelligence* model decreased the mean time required to reach a diagnosis by 15.0% and 23.7% for the first and second pathologists, respectively. On analyzing digital slides, the tool was effective in supporting human diagnostic thinking.

**Conclusions:**

The Domain-KEY algorithm successfully mimicked human decision-making skills and supported expert pathologists in the routine diagnosis of PNI.

## Introduction

Despite decades of intense scientific exploration, oral cavity squamous cell carcinoma (OCSCC) remains a major public health concern with significant costs to patients and their families – especially in South Central Asia and Pacific Islands where betel nut chewing is endemic (1, 2). While surgical resection remains the mainstay of treatment, a multimodal approach to therapy is increasingly being used to treat advanced tumors (3–6). Therefore, there is an urgent, unmet need to develop accurate risk stratification tools specifically designed to identify patients who are most likely to benefit from adjuvant therapy. A detailed analysis of pathological risk factors (RFs) is also necessary to improve adjuvant treatment tailoring (7) and weighted risk score systems with this aim have been proposed (8).

Perineural invasion (PNI), a form of local invasion defined as the ability of cancer cells to invade in, around, and through nerves, poses an indication for adjuvant treatment and has a negative prognostic impact (9, 10). Unfortunately, the diagnosis of PNI suffers from a significant degree of intra- and inter-observer variability. The use of deep learning (DL) techniques and artificial intelligence (AI) holds promise to overcome these limitations (11–18). In recent years, there have been several attempts in the field of digital pathology to comprehensively capture the histology features of OCSCC (19, 20). While current DL-based algorithms are generally fully-automated, their robustness is largely dependent on the availability of big data sources. In this scenario, a human-AI interaction approach can enhance the accuracy and efficiency of pathological readings to render accurate diagnoses. The necessity of this alliance under the framework of *hybrid intelligence* is necessary for the reliability of tasks based on relatively limited training datasets, as those available for pathological research. Starting from these premises, the aim of this study was to develop a DL-based human-enhanced tool for identifying PNI in digital slides obtained from patients with OCSCC. On the one hand, such an approach enables a more objective pathological assessment of PNI that is suitable for clinical prognostication. On the other hand, our tool has the potential to tailor treatment at the individual level.

## Materials and methods

### Ethical statement

Pathology slides of OCSCC were obtained from the Tissue Bank of the Chang Gung Memorial Hospital, Linkou (Taoyuan, Taiwan). The local Institutional Review Board (identifier: 2020-00-346B) granted ethical approval to process and analyze all data.

### Study patients

The study cohort consisted of 80 patients with first primary OCSCC (76 men and 4 women; mean age: 56 years; age range: 36−82 years; **Table 1**) enrolled from 2011 to 2017. The occurrence of PNI increased in a stepwise fashion according to the pT status, as follows: pT1, 6.2%; pT2, 22.7%; pT3, 34.2%; and pT4, 47.3%.

**Table 1:** General characteristics of patients with oral cavity squamous cell carcinoma included in the study.

Characteristic (n, %; before PS matching)	n	%
Sex		
Men	76	95.0
Women	4	5.0
Age (years)		
Range: 36−82		
Mean: 56		
Tumor subsite		
Tongue	40	50.1
Buccal	20	25.0
Gum	9	11.3
Floor of mouth	5	6.3
Retromolar trigone	4	5.0
Lip	2	2.5
Pathologic T status		
T1	2	2.5
T2	33	41.3
T3	3	3.8
T4	42	52.6
Pathologic N status		
pN0	26	32.5
pN1	5	6.3
pN2	28	35.0
pN3b	21	26.3
Pathologic stage		
I	2	2.5
II	19	23.8
III	5	6.3
IV	54	67.5

### Validation cohort

The predictive accuracy of the Domain-KEY algorithm was validated by taking into account a total of 25 whole-slide images (WSIs) obtained from seven patients with oropharyngeal (cancer of the tongue base, n = 1; tonsil cancer, n = 1; soft palate cancer, n = 1) and hypopharyngeal (cancer of posterior wall, n = 2; pyriform sinus cancer, n = 2) malignancies.

### Image acquisition and ground truth annotation

**Figure 1:**
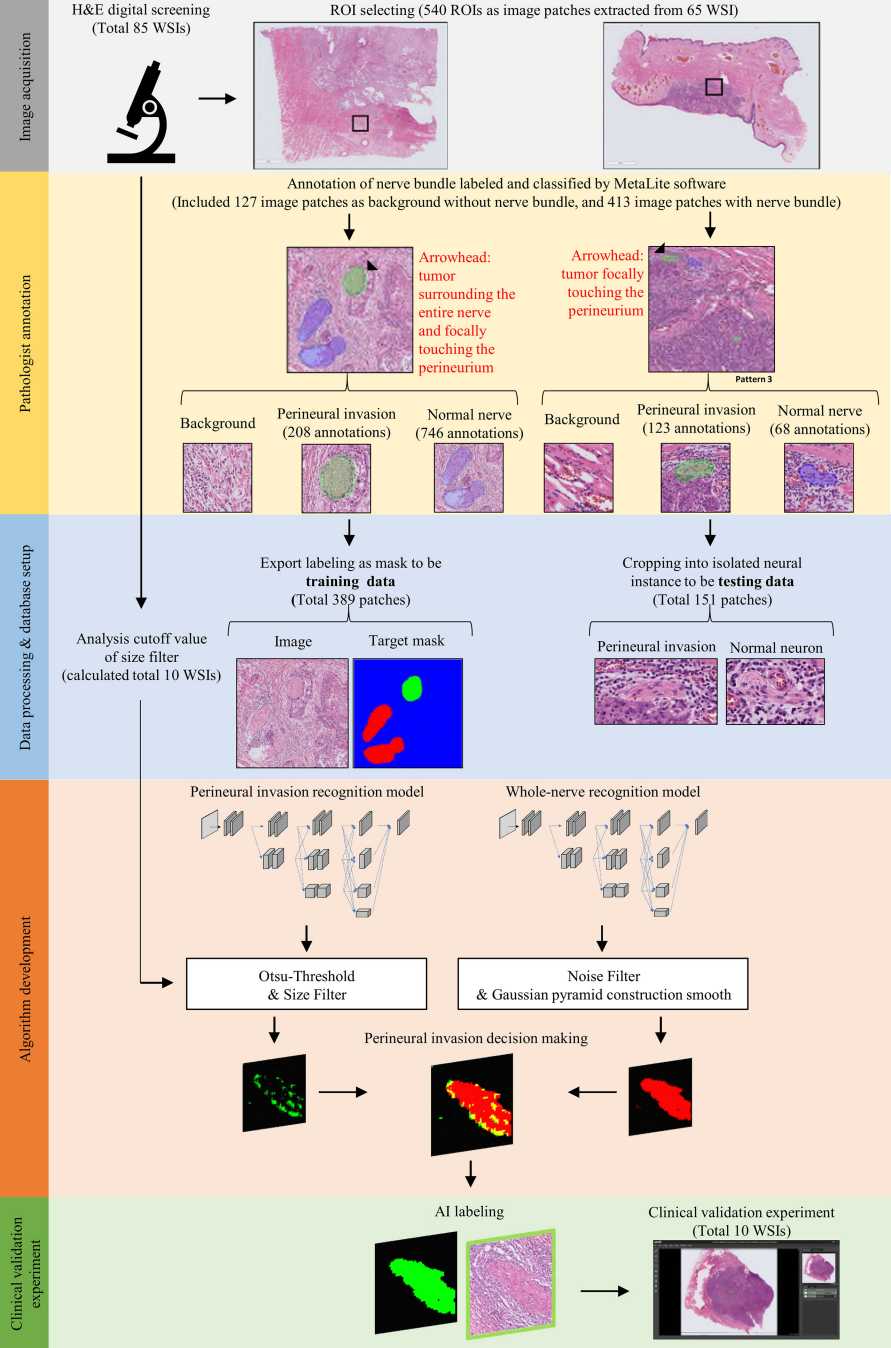
Workflow used for image acquisition and ground truth annotation. Image acquisition was undertaken by selecting and extracting ROI patches (black rectangle) from whole slide images. Subsequently, trained technicians supervised by an expert pathologist annotated areas of perineural invasion (marked in green) and normal nerve structures (marked in blue). Finally, labeled patches were processed to binary training data sets with appropriate masks; alternatively, they were used for building a testing data set for the recognition of nerve structures.

**Figure 1** provides a workflow diagram for ground truth image annotation. Hematoxylin and eosin (H&E)-stained tissue slides (n = 85) without pen marks were obtained from all study participants. A NanoZoomer S360 scanner (Hamamatsu Photonics; Hamamatsu City, Japan) was used to acquire (400× magnification) and export (NDPI file format; size: 0.23 × 0.23 micron per pixel) WSIs (n = 85). A total of 540 regions of interest (ROIs) were manually selected from 65 WSIs (mean size: 5000 × 5000 pixels) and the resulting images were stored (Tag Image File Format [TIFF]) using the scanner’s built-in software. The remaining 20 WSIs were used to develop the decision rule (n = 10) and validation of the prediction (n = 20). On analyzing the 540 TIFF images with the MetaLite software, we identified 1145 nerves, of which 814 were normal and 331 had evidence of PNI. A total of 127 TIFF images were selected as background through the exclusion of the region used for ground truth labeling. Ground truth annotation was performed in MetaLite by adding specific labeling layers. All procedures were independently performed by three trained technicians supervised by an experienced pathologist. Since the analysis was undertaken by independent assessors, overlaps of structures labeled as PNI or normal nerve within the same patch were possible. TIFF files (n = 540) containing ROIs were split into training (n = 389) and testing (n = 151) sets. The latter was dichotomized and used to test the presence of PNI (n = 94) *versus* normal nerve (n = 57). **Table 2** and **Figure 1** provide a summary of data preparation.

**Table 2:** Summary of image acquisition and data preparation.

Dataset name	Whole slide images, n (NDPI format)	Image patches, n (TIFF format)	Ground truth annotation, n
Background^*^	39	127	127
Nerve identification feature^*^	53	148 (perineural invasion)^†^	331 (perineural invasion)
		398^†^ (normal nerve)^†^	814 (normal nerve)
Size filter	10	–	–
Clinical validation	10	–	–
Total number, n	85	540 (389 used for training and 151 for testing [57 for nerve fibers and 94 for perineural invasion])	1272

NDPI, NanoZoomer Digital Pathology Image; TIFF, Tagged Image File Format.

^*^A portion of the whole-slide image was used for creating both the background and nerve identification datasets.

^†^Since the analysis was undertaken by independent assessors, overlaps of structures labeled as perineural invasion or normal nerve within the same patch were possible.

### MetaLite annotation software

TIFF files were annotated using the MetaLite open-source software tool. Differently from similar open-source image analysis programs for digital pathology, MetaLite provides a simple install wizard and has a user-friendly interface. Furthermore, it supports, reads, and writes the common image formats used in digital pathology and the Digital Imaging and Communications in Medicine (DICOM) standard. MetaLite offers several tools (e.g., brushes, erasers, and polygons) that can be used to annotate images using keyboard shortcuts. It also offers an AI plug-in that enables the training of machine learning models. The source code is available for use, modification, and distribution with its original rights (https://github.com/JelloXBiotechInc/MetaLite).

### Model architecture and algorithm development

**Figure 2:**
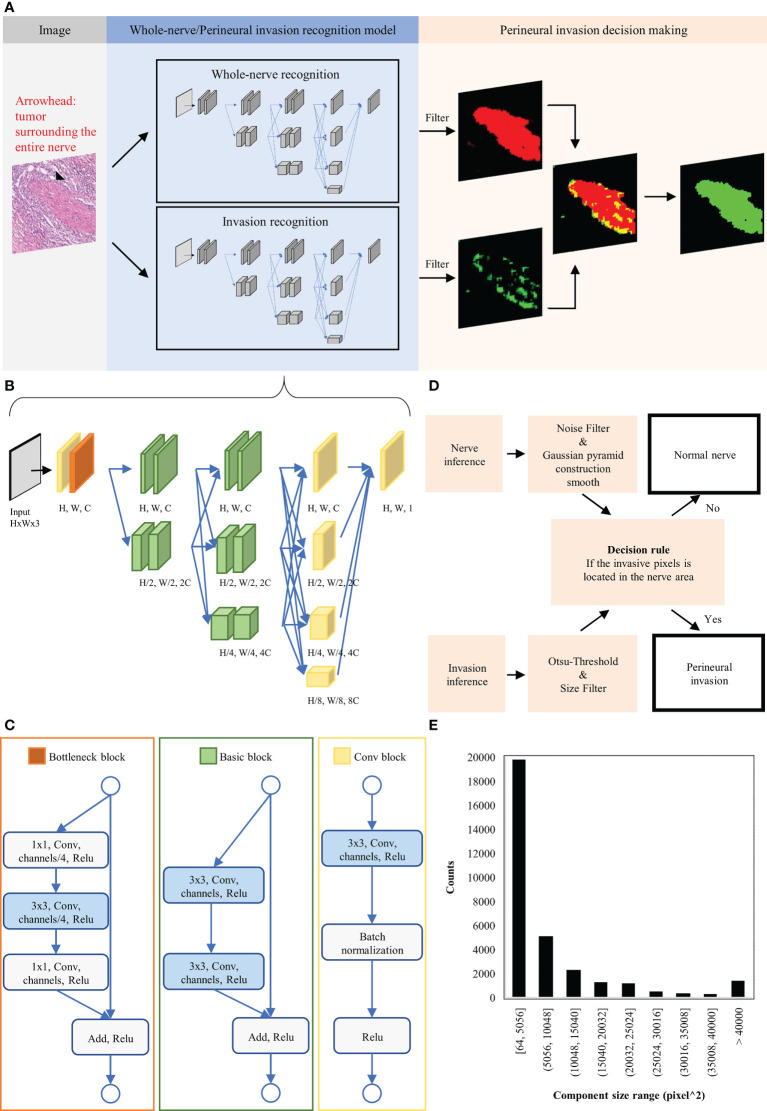
Schematic representation of the domain knowledge enhanced yield (Domain-KEY) algorithm applied to identify perineural invasion. **(A)** Workflow for algorithm development. **(B)** Architecture of the deep learning image segmentation model used to identify normal nerve structures and perineural invasion. **(C)** Detailed description of the function block architecture. **(D)** Creation of a rule flow for identifying perineural invasion. Inferred results from the identification of normal nerve structures and perineural invasion underwent post-processing and irregular components were filtered out. Each nerve structure was subsequently inspected for the presence or absence of perineural invasion. **(E)** Distribution of the invasive component outside the nerve structure. These findings were considered as noisy signals and used to choose filters of appropriate sizes.

For the purpose of this study, PNI was defined as tumor cell invasion in, around, and through the nerves (21, 22). A domain knowledge-enhanced yield (Domain-KEY) algorithm for the detection of PNI was developed as a DL-based human-enhanced tool. A schematic flowchart of the algorithm is depicted in **Figure 2A**. The model structure consists of two parts to simulate human decision-making skills in diagnostic pathology. The first part comprised two semantic segmentation models – one aimed at recognizing the presence of nerve fibers and the other one at identifying PNI. The inferred results were subsequently subjected to post-processing of generated decision rules to make a diagnostic decision. The DL architecture was applied to recognize the presence of nerve fibers and subsequently identify PNI. Using a benchmark model for segmentation (High-Resolution Network, version 2) (23), four multi-resolution group convolutions were designed by connecting high-to-low resolutions to recover a high-resolution representation. Multiple representations were also mixed in the final stage of the process to achieve the target resolution. This approach allowed extracting feature information at micro and macro levels from different resolutions and achieved qualified accuracy in a pilot training set. The network ranged from 256 (H) × 256 (W) × 3 to 256 (H) × 256 (W) × 1. The basement channel (C) was equal to 32. The resolution in the function decreased using the convolution with stride 2 and increased using bilinear up-sampling. The architecture of the model is shown in **Figure 2B**. The network structure consists of three parts termed A, B, and C (**Figure 2C**). Part A comprised a convolutional block (i.e., the basic network unit composed of a convolutional layer), a batch normalization layer, and a rectified linear unit (ReLU) activation function. Part B consisted of a bottleneck block to maintain spatial resolution and enlarge the receptive field. Finally, part C was a basic block aimed at maintaining low-level features.

The network model was developed on an NVIDIA GTX 2080 Ti Graphics Card using the TensorFlow architecture and subsequently trained with the Adam algorithm for optimization (initial learning rate: 0.001). During data augmentation to increase the dataset size by a magnitude of 2048, training pairs (image and target mask) were randomly cropped, sliced, and rescaled (size: 256 × 256). Training data were also augmented by flipping each image along the horizontal and vertical axes. Stain variations and noise were minimized to improve the stability of the model. With this aim, we randomly adjusted brightness [-30%, +30%], saturation [0.7, 1.3], and contrast [0.7, 1.3] of each image. The training process converged when the mean square error loss for recognizing the presence of nerve fibers and PNI fell to 0.0082 and 0.0077, respectively. The value range of RGB channels was then normalized to [0, 1] to speed up learning and improve performance.

The diagnostic workflow for the presence of PNI is summarized in **Figure 2D**. The inference results for the identification of nerve fibers were initially processed with a Gaussian pyramid. A threshold filter and a smoothing construction algorithm were applied to reduce noise and the unusual right angle observed for nerve fibers. The inference results for the diagnosis of PNI were processed by combining constant thresholding and the Ostu’s thresholding technique to filter signals undetectable to the human eye. A size filter was also applied to remove irregular components that increased noise. The optimal cutoff for this filter was identified by inferring the presence of nerve fibers and the perineurium on ten H&E-stained digital slides. A derived signal to identify PNI was then calculated based on 1) the inferred presence of nerve fibers and the perineurium and 2) the presence of irregular components. We then examined the size of irregular components to rule out their origin from noisy image segmentation (**Figure 2E**). On analyzing five WSIs, we identified 30 cells responsible of PNI with a mean size of 13.5 µm; this finding is in line with the diameter of malignant squamous cell carcinoma cells (mean: 13 ± 2 µm) (24, 25). The analysis of size distribution revealed that part of the irregular components had a size > 40,000 pixel^2 (i.e., 14−15 cells). Filtering of all irregular components was deemed to render the algorithm less sensitive as a result of an erroneous exclusion of certain PNI foci. At 10% and 5% levels of significance (26), the cutoff values were 21,632 pixel^2 and 36,864 pixel^2, respectively. As the latter cutoff was close to 40,000 pixel^2, we decided to apply a 10% level of significance and use a value of 21,632 pixel^2 to filter out 90% of the irregular components. Therefore, the detection limit of the algorithm was equal to ~8 cells.

**Figure 3:**
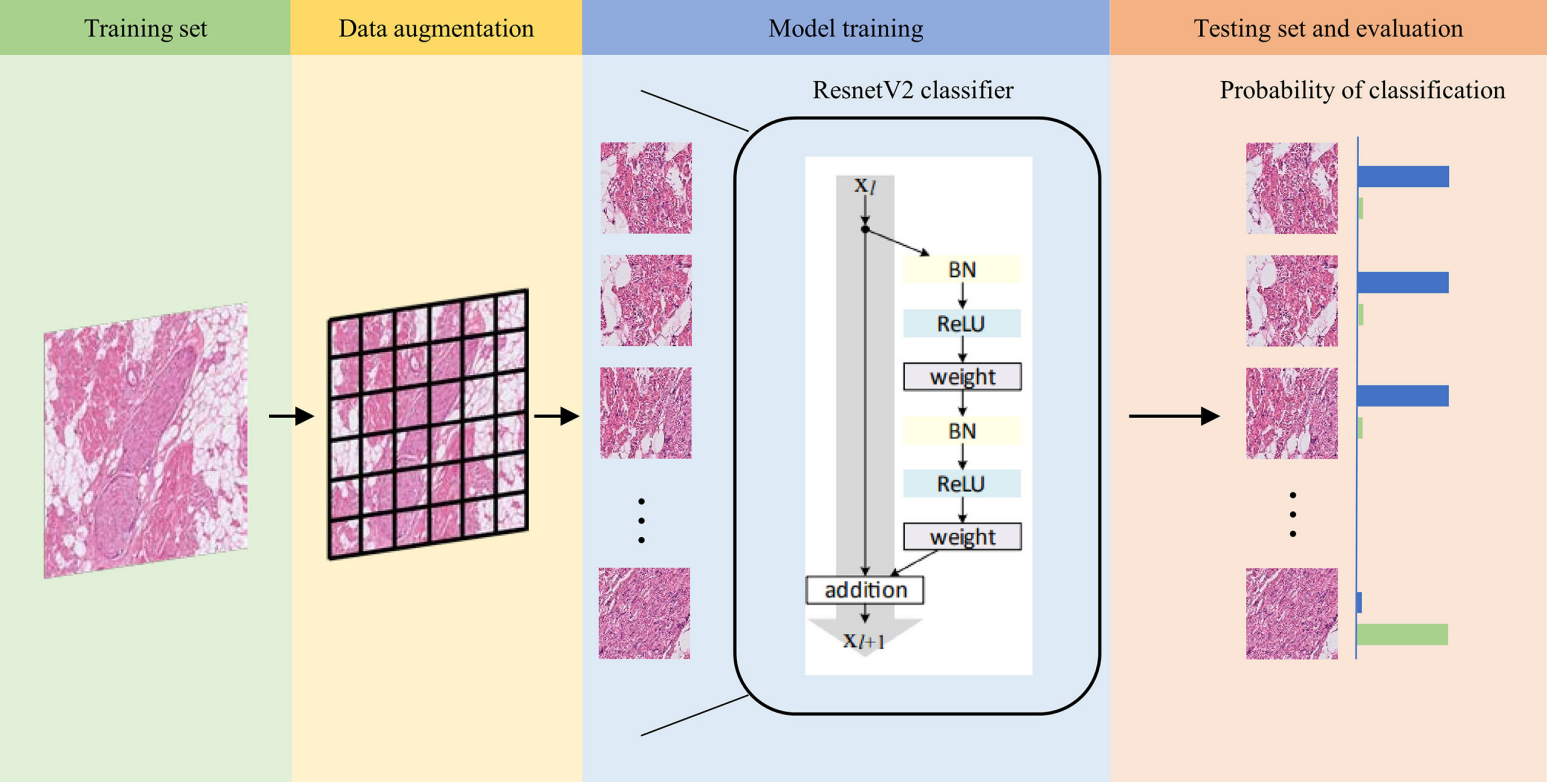
Workflow used for training and testing the ResnetV2_50 reference model. Images in the training set (green background) were split into smaller patches for data augmentation (yellow background) and subsequently trained with the ResnetV2 model (blue background). When the model converged to a good training accuracy, test results were calculated (orange background). The trained model was finally applied to analyze patches and assign a probability of normal nerve fibers (blue bar) and perineural invasion (green bar). When the probability of normal nerve fibers was higher than that of perineural invasion, the image was labeled as normal nerve (and vice versa).

We next examined the performance of the Domain-KEY algorithm for the diagnosis of PNI. With this aim, a workflow based on DL technology was implemented without post-processing and decision thinking. The ResNet_v2_50 model was used to train and test the same dataset for reference purposes (27). The process of model training and testing is summarized in **Figure 3**. In the data preprocessing phase, a total of 389 ROIs were split into smaller patches (1024 ×1024 pixels, n = 6281) for training. Furthermore, 94 ROIs were split into 1729 patches for testing. The ResNet_v2_50 classification model was applied as a feature extractor and fully connected to an output dense layer 2. The kernel method was used for feature extraction from the existing pre-trained model (http://www.tfhub.dev/google/imagenet/resnet_v2_50/feature_vector/5). The output layer was initialized with a random normal distribution. During the training progress, the weight kernels were updated by the Adam Optimizer with an initial learning rate of 0.001 and an exponential decay function was applied every 50000 steps. The model was trained with a batch size of 10. Each image was resized to 224 × 224 and randomly flipped along the horizontal and vertical axes. When the mean Softmax cross entropy loss fell to 0.001, the training process converged. During testing, splits from ROI images were inferred by the trained model to assign a diagnosis of PNI (presence *versus* absence).

### Validation of the hybrid intelligence model: Diagnostic time analysis

A total of 10 H&E-stained WSIs used for the development of the decision rule were employed to validate the *hybrid intelligence* model by taking into account the time required to reach a diagnosis. Label-free WSIs underwent visual inspection by two independent pathologists (LYL and CHY) to assess the PNI status; in parallel, the diagnostic time for each slide was carefully annotated. After a washout interval of at least 8 weeks, the same WSIs were re-inspected after the addition of labels provided by the *hybrid intelligence* model. The time required to reach a diagnosis was then recorded for comparison purposes.

## Results

### Identification of nerve fibers

**Figure 4:**
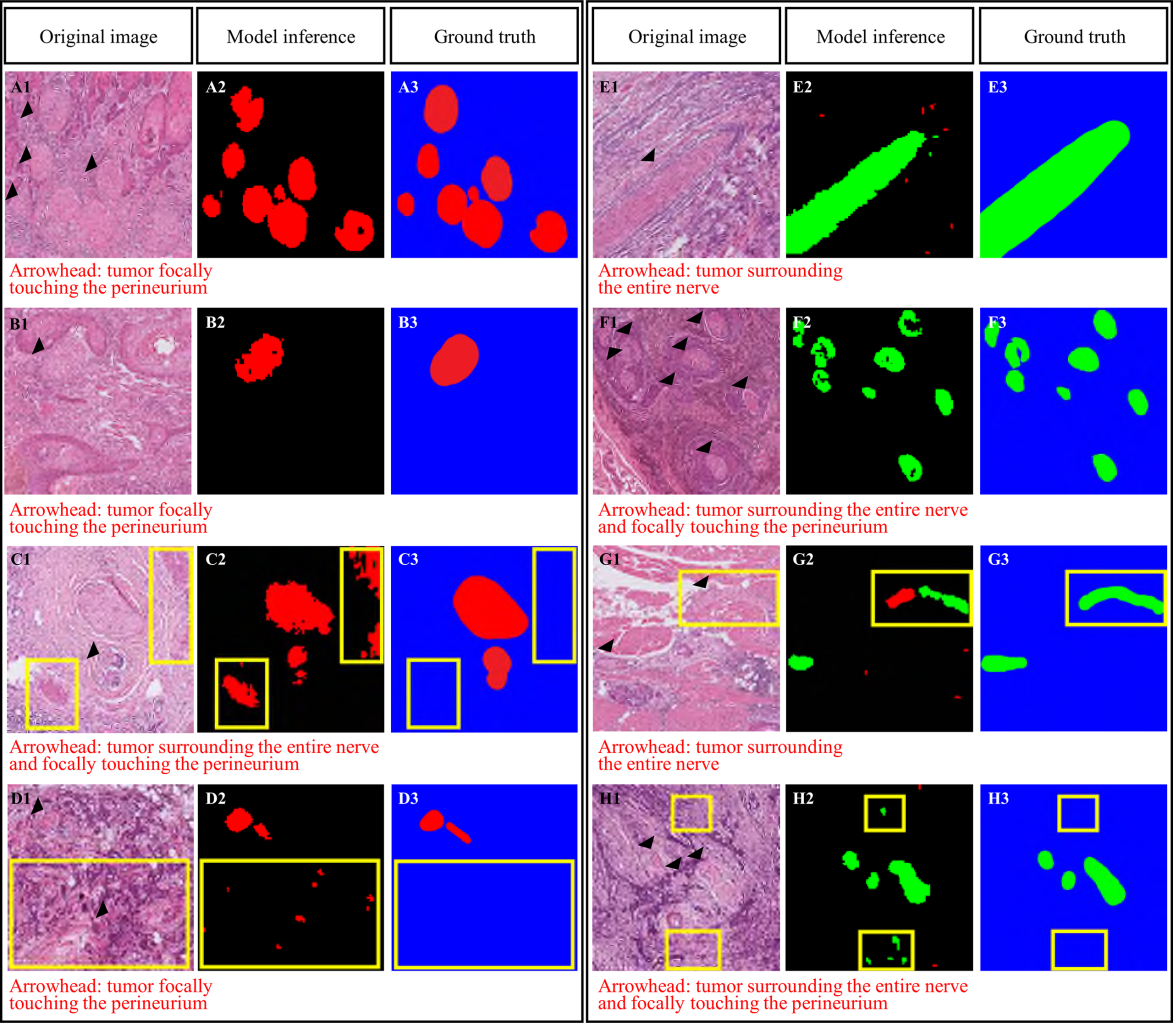
Domain knowledge enhanced yield (Domain-KEY)-based classifier for the presence of perineural invasion. Correct **(A, B)** and incorrect **(C, D)** identification of nerve fibers. Incorrect classification resulted from the presence of similar patterns **(C)** or noisy signals **(D)**. Correct **(E, F)** and incorrect **(G, H)** identification of perineural invasion. Incorrect classification resulted from the presence of fibroblasts surrounding the nerve and inappropriate separation of nerve fibers **(G)** or noisy signals **(H)**. The purple color denotes the recognition of nerve structures, whereas normal nerves are marked in red. Areas of perineural invasion are highlighted in green with a black/blue background. The yellow rectangle denotes an area of incorrect classification.

The sensitivity, specificity, and accuracy of the DL-based Domain-KEY algorithm for detecting nerve fibers were 92.98%, 83.33%, and 91.67%, respectively (**Table 3**). Specificity was lower than sensitivity, thereby suggesting that the algorithm would produce more false positives than false negatives. A graphic depiction of true positives, true negatives, false positives, and false negatives is shown in **Figures 4A−D**. Images with misclassification errors were reviewed by two pathologists (LYL and CHY). The results revealed that the presence of fibroblasts and inflammatory cells surrounding adjacent nerves sporadically resulted in classification errors.

**Table 3:** Confusion Matrix and Testing Performance of the Model for Nerve Identification (Number of Nerve Structures, n = 132).

	Actual: positive (*n*)	Actual: negative (*n*)	
Predicted: positive	TP (106)	FP (3)	
Predicted: negative	FN (8)	TN (15)	
	Sensitivity: 92.98%	Specificity: 83.33%	Accuracy: 91.67%

TP, true positive; FP, false positive; FN, false negative; TN, true negative.

### Diagnosis of perineural invasion

**Figure 5:**
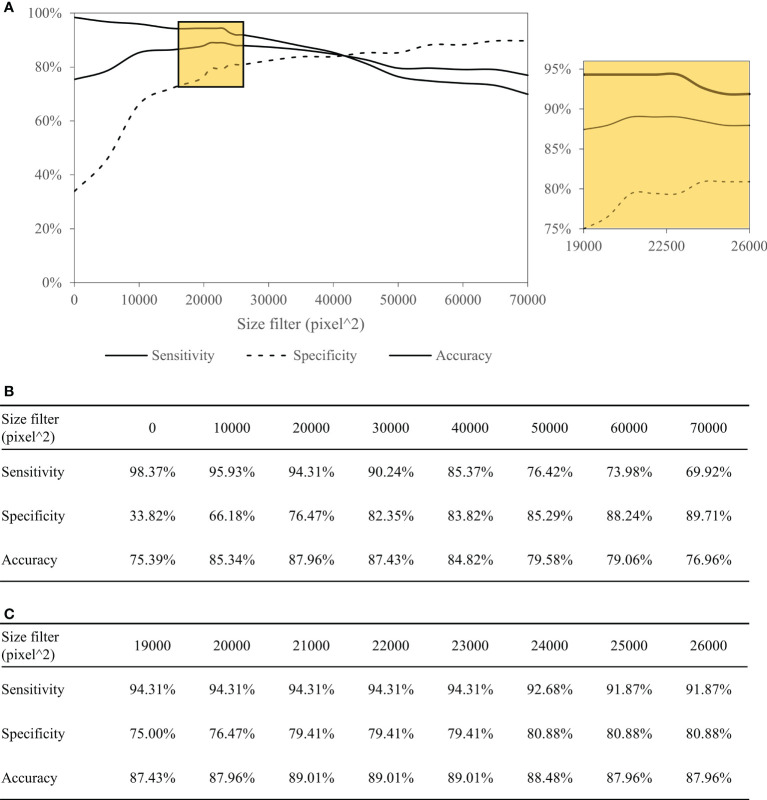
Performance of the Domain-KEY algorithm following the application of various size filters with different cutoffs. Curves depicting the diagnostic performance **(A)** as well as sensitivity, specificity, and accuracy **(B, C)** of the Domain-KEY algorithm following the application of various size filters with different cutoffs. The cutoff values started from 0 pixel^2 and were increased in stepwise fashion using an interval of 10,000. The accuracy for the detection of PNI improved by 9.95% when the cutoff increased from 0 to 10,000 pixel^2, with an optimal diagnostic performance (>87%) in the 20,000−30,000 pixel^2 range. When the cutoff was restricted to a 21,000−23,000 pixel^2 range, an accuracy >89% was achieved. When cutoff values >30,000 pixel^2 were tested, the accuracy decreased and subsequently fell to values similar to those observed when a cutoff of 0 pixel^2 was used.

**Table 4** summarizes the performance of the DL-based Domain-KEY algorithm for the diagnosis of PNI. Our algorithm (191 testing levels) markedly outperformed the reference ResnetV2_50 model (1729 small patches). The sensitivity, specificity, and accuracy for the detection of PNI were 94.31% *versus* 35.03%, 79.41% *versu*s 69.37%, and 89.01% *versus* 61.94%, respectively. The most marked improvement occurred in terms of sensitivity, as a result of the pixel-wise resolution of semantic segmentation. A graphic depiction of true positives, true negatives, false positives, and false negatives is shown in **Figures 4E–H**. The algorithm easily identified even small areas of PNI. However, the pixel-wise resolution resulted in noisy signals originating from small-sized objects. In addition, certain nerves were sporadically separated into different components. Since this lowered the specificity of the algorithm to some extent, various size filters were applied. **Figure 5** shows how sensitivity and specificity varied according to the filter size. The accuracy for the detection of PNI improved when the cutoff increased from 0 to 10,000 pixel^2, with an optimal diagnostic performance in the 20,000−30,000 pixel^2 range. When the cutoff was restricted to a 21,000−23,000 pixel^2 range, an accuracy >89% was achieved. When cutoff values >30,000 pixel^2 were tested, the accuracy decreased and subsequently fell to values similar to those observed when a cutoff of 0 pixel^2 was used. Collectively, these results indicate that the inclusion of a size filter into the algorithm is appropriate.

**Table 4:** Confusion matrix and testing performance of the model for identifying perineural invasion (Regions of Interest), Domain-KEY AI model *versus* ResnetV2_50 model.

**(A) CGMH method (Domain-KEY AI) (number of nerve structures, n = 191)**			
	Actual: positive (*n*)	Actual: negative (*n*)	
Predicted: positive	TP (116)	FP (14)	
Predicted: negative	FN (7)	TN (54)	
	Sensitivity: 94.31%	Specificity: 79.41%	Accuracy: 89.01%
			
**(B) ResnetV2_50 (number of patches, n = 1729)**			
	Actual: positive (*n*)	Actual: negative (*n*)	
Predicted: positive	TP (64)	FP (246)	
Predicted: negative	FN (310)	TN (1109)	
	Sensitivity: 35.03%	Specificity: 69.37%	Accuracy: 61.94%

CGMH, Chang Gung Memorial Hospital; AI, artificial intelligence; TP, true positive; FP, false positive; FN, false negative; TN, true negative.

### Integration of the domain-KEY algorithm in digital pathology

**Figure 6:**
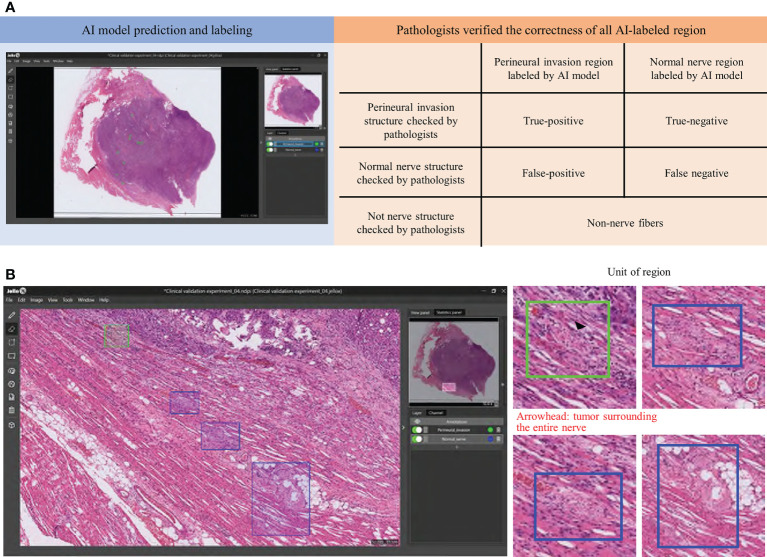
A *hybrid intelligence* model was developed to integrate human decision-making in clinical pathology with data extracted by the Domain-KEY algorithm. Two pathologists checked the correctness (orange background) of all Domain-KEY-labeled (blue background) whole-slide images **(A)**. The review was implemented after the integration of the Domain-KEY AI algorithm into an open-source software (MetaLite). Green and blue rectangles denote Domain-KEY-labeled areas of perineural invasion and normal nerve fibers, respectively **(B)**.

Clinical digital pathology is generally based on the assessment of WSIs rather than ROI patches. To accelerate the integration of the Domain-KEY algorithm into routine practice, the tool was plugged into the MetaLite software and run on WSIs. Pathologists were therefore able to open digital WSIs and execute the plug-in to add AI-annotated masks for detecting nerve fibers and identifying PNI (**Figure 6**). The *hybrid intelligence* diagnostic framework was implemented as follows. First, ten H&E-stained WSIs not previously used in the study were read and labeled by the Domain-KEY algorithm. Thereafter, the labeling correctness was visually inspected by two independent pathologists (LYL and CHY). They initially checked whether the area included in the rectangle consisted of nerve fibers. Subsequently, a quality control of the AI-based prediction model for the presence of PNI (green rectangle area) *versus* normal nerve fibers (blue rectangle area; **Figure 6B**) was undertaken.

### Accuracy of the hybrid intelligence model for detecting nerve fibers

After quality control of >400 labels, the mean accuracy of the model for the presence of nerve fibers was 87.90% and 87.09% for the first and second pathologists, respectively (mean accuracy: 87.50%, **Table 5**). These values were lower than the accuracy found in the testing set of ROI images; this was caused by differences in sensitivity values (75.00% *versus* 91.66% for the first and second pathologists, respectively) related to the different number of false negatives.

**Table 5:** Confusion matrix and performance of the model used for pathological validation of perineural invasion (whole slide images).

First pathologist (LYL)*			
	Actual: positive (*n*)	Actual: negative (*n*)	
Predicted: positive	TP (12)	FP (7)	
Predicted: negative	FN (4)	TN (384)	
	Sensitivity: 75.00%	Specificity: 98.20%	Accuracy: 97.29%
			
Second pathologist (CHY)*			
	Actual: positive (*n*)	Actual: negative (*n*)	
Predicted: positive	TP (11)	FP (8)	
Predicted: negative	FN (1)	TN (385)	
	Sensitivity: 91.66%	Specificity: 97.96%	Accuracy: 97.77%

TP, true positive; FP, false positive; FN, false negative; TN, true negative.

*Accuracy for recognition of nerve structures: First pathologist, 87.90% (407/463); Second pathologist, 87.09% (405/460).

### Accuracy of the hybrid intelligence model for detecting perineural invasion

The mean accuracy for the diagnosis of PNI increased from 89.01% to 97.53% after pathology assessment. This was caused by the presence of invasive features (identified in 8 out of 12 cases) that were easily identified by the pathologists. This value was in line with the accuracy found in the testing set of ROI images.

### Accuracy in a validation dataset of oropharyngeal and hypopharyngeal tumors

**Figure 7:**
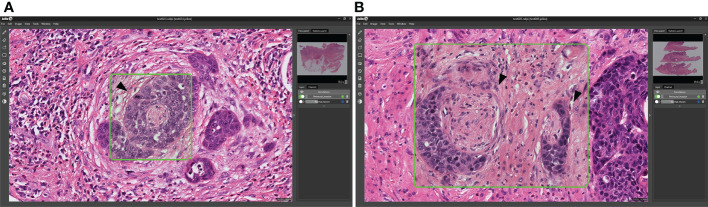
Validation of the Domain-KEY algorithm for identifying PNI in oropharyngeal and hypopharyngeal malignancies. **(A)** Identification of PNI in oropharyngeal carcinoma (green box). The arrowhead indicates the tumor surrounding the entire nerve **(B)**. Identification of PNI in hypopharyngeal carcinoma (green boxes). The arrowhead indicates the tumor that focally touched the perineurium.

All of the 25 images obtained for patients with either oropharyngeal and hypopharyngeal tumors contained PNI, which was successfully identified by the Domain-KEY algorithm in 24 cases (accuracy = 96%; **Figures 7A, B**). Therefore, the observed accuracy in the validation dataset was similar to that originally observed for OCSCC (97.5%).

### Added diagnostic value of the hybrid intelligence model: Diagnostic time reduction

On analyzing ten WSIs, the addition of the *hybrid intelligence* model decreased the mean time required to reach a diagnosis by 15.0% and 23.7% for the first and second pathologist, respectively (**Table 6**). In one case, the algorithm was also able to detect previously unidentified PNI features.

**Table 6:** Impact of the model in decreasing the time required to reach a diagnosis: analysis of whole-slide images.

First pathologist (LYL)				
Whole-slide image identifier	Diagnostic time without the application of Domain-KEY AI, sec	Diagnostic time with the application of Domain-KEY AI, sec	Percentage change in diagnostic time	Notes
S2012-xxx201	37	126	-240.5%	
S2018-xxx884	50	89	-78.0%	
S2018-xxx153	32	22	31.3%	
S2018-xxx810	54	19	64.8%	
S2019-xxx702	70	131	-87.1%	
S2019-xxx637	160	75	53.1%	
S2020-xxx911	90	44	51.1%	
S2020-xxx163	30	26	13.3%	
S2021-xxx140	77	49	36.4%	
S2021-xxx542	108	21	80.6%	
Total time, sec	708	602	15.0%	
Second pathologist (CHY)				
S2012-xxx201	20	72	-260.0%	
S2018-xxx884	45	32	28.9%	
S2018-xxx153	55	14	74.5%	
S2018-xxx810	175	12	93.1%	Initially, the pathologist did not identify PNI; however, the decision was reversed based on the algorithm’s results
S2019-xxx702	55	131	-138.2%	
S2019-xxx637	101	110	-8.9%	
S2020-xxx911	51	98	-92.2%	
S2020-xxx163	36	12	66.7%	
S2021-xxx140	42	57	-35.7%	
S2021-xxx542	138	10	92.8%	
Total time, sec	718	548	23.7%	

AI, artificial intelligence; PNI, perineural invasion.

## Discussion

In this pilot study, we described a robust DL-based human-enhanced tool for identifying PNI in digital slides obtained from patients with OCSCC. Our results indicated that pathologists can reach an accurate diagnosis of PNI with the aid of the Domain-KEY algorithm (accuracy values for ROI patches and WSIs: 89.01% and 97.53%, respectively). Notably, the classification accuracy provided by our model is not only higher than those of current algorithms used to perform object detection but it is also in line with the values reported by recent AI-based technologies in diagnostic pathology. (20, 24) An inherent advantage of our algorithm is that it can be integrated as a plug-in into the MetaLite open-source software to meet the growing need for user-friendly tools in digital pathology.

While the pixel-wise resolution of the Domain KEY algorithm allowed detecting the presence of PNI at a fine level of detail, noisy signals originating from small-sized objects were a potential source of confounding. This issue was addressed with the application of a size filter (cutoff: 21,632 pixel^2) during the post-processing phase. Under these circumstances, the limit of detection of our algorithm was of approximately 8 cells.

It can be argued that this approach can lead to misclassification when small clusters of tumor cells are invading the nerve. However, on assessing the presence of PNI, the pathologist’s attention is chiefly focused on the presence of tumor cell nests rather than isolated malignant cells. In addition, an analysis of diagnostic accuracy carried out on ROI patches and WSIs revealed that a satisfactory performance for our algorithm. The Domain-KEY algorithm described in our study outperformed the ResnetV2_50 classifier for the detection of PNI (diagnostic accuracy: 89.01% and 61.94%, respectively). In addition, we implemented a comparison between the Domain-KEY algorithm and the HRNet_v2 model, which relied on a similar architecture (semantic segmentation) for the detection of PNI in the absence of neural recognition. Despite a slight improvement compared with the ResnetV2_50 classifier, the diagnostic accuracy of the HRNet_v2 model was still lower (63.87%) than that of the Domain-KEY algorithm (**Supplementary Table 1**). Collectively, these results indicate that the Domain-KEY algorithm markedly outperforms the direct use of DL technologies. This is attributable to the two-step recognition workflow implemented in the Domain-KEY model. Specifically, our algorithm initially detects the presence of nerve structures (first step) followed by the specific identification of PNI (second step). This stepwise approach was found to outperform in terms of diagnostic accuracy other predictive models that rely on 1) high-resolution semantic segmentation for identifying PNI at a fine level of detail and 2) the use of size filters to reproduce the pathologist’s thinking (which is chiefly focused on the presence of tumor cell nests rather than isolated malignant cells). On analyzing WSIs obtained from patients with OCSCC, our algorithm was able to achieve a mean diagnostic accuracy as high as 97.5% after image review carried out by two pathologists. Notably, the Domain-KEY algorithm showed a similar accuracy (96%) in detecting PNI in a validation data set of 25 WSIs obtained from seven patients with oropharyngeal and hypopharyngeal malignancies.

A significant added value of the Domain-KEY algorithm was the reduction in the time required to reach a diagnosis when WSIs were analyzed. Moreover, our tool allowed detecting previously unidentified PNI in digital slides. Collectively, these preliminary results indicate that the Domain-KEY algorithm has the potential to improve routine pathology practice in the assessment of PNI. However, the presence of noisy signals that may lead to diagnostic artefacts should be acknowledged as a significant shortcoming. Additional optimization is therefore necessary before routine application of the proposed technique.

Notably, our validation experiments were designed to mimic the PNI rates observed in clinical practice. In this scenario, WSIs contained both normal nerves and areas of PNI, although infiltration of nerves by cancer cells occurred rarely. Since true positive and false positive rates were similar, the precision rate of the algorithm decreased in parallel. Although OCSCC offers a unique opportunity to develop and validate novel DL algorithms for digital pathology, AI-assisted approaches have been so far rarely applied to this malignancy. Published studies in the field have been limited to the detection of primary tumor cells using conventional support vector machine learning algorithms or convolutional neural network-based DL techniques (**Table 7**). This is, to our knowledge, the first attempt to identify PNI in OCSCC slides through an AI-based classifier. A correct diagnosis of PNI is more challenging than the identification of primary tumor cells. For this reason, our algorithm was designed to mimic human decision-making skills through a stepwise approach (i.e., identification of nerve fibers followed by the diagnosis of PNI). Since PNI has adverse prognostic implications in different solid malignancies (e.g., pancreatic cancer, colorectal cancer, gastric cancer, prostate cancer, and other head and neck tumors) (28), our approach may have broader clinical applications.

**Table 7:** Published studies focusing on computer-assisted pathological diagnosis for oral cavity squamous cell carcinoma.

Authors [reference] (year of publication)	Cancer site	Endpoint	Dataset	Methods	Accuracy	Accuracy (pathological validation based on slide examination)
Lee et al. [Current study]	Oral cavity	Identification of nerve structures and diagnosis of perineural invasion in oral cavity SCC	65 WSIs, 540 patches (331 annotation for perineural invasion, 814 for normal nerve), 10 WSIs for pathological validation	Domain knowledge enhanced yield (Domain-KEY) algorithm as a form of hybrid intelligence to identify nerve structures and diagnose perineural invasion in oral cavity SCC.	89%	97.5%
Das et al. (20)	Oral cavity	Benign lesions; SCC with different tumor differentiation levels (well, moderately, and poorly differentiated)	156 WSIs, 8321 patches	CNN based multiclass grading classifier for automated classification of differentiation levels in oral cavity SCC.	97.5%	–
Halicek et al. (24)	Head and neck	Benign lesions; SCC	228 WSIs (head and neck) 124 WSIs (oral cavity)	Two-dimensional CNN classifier based on the Inception V4 architecture for predicting the probability of cancer on analyzing segmented patches from WSIs.	85%* (AUC: 0.916)	- (AUC: 0.944)
Rahman et al. (19)	Oral cavity	Benign lesions; SCC	42 WSIs, 476 patches (237 benign lesions, 483 SCC)	SVM classifier for automated binary classification of oral cavity SCC based on texture features.	100%	–

SCC, squamous cell carcinoma; WSI, whole-slide image; CNN, convolutional neural network; SVM, support vector machine; AUC, area under curve.

*Data from head and neck squamous cell carcinoma.

Apart from PNI, both lymphatic invasion and vascular invasion have been associated with an increased risk of local recurrence, lymph node metastasis, distant metastasis, and less favorable survival figures in patients with OCSCC (8). On analyzing a large cohort of 1570 patients with first primary OCSCC diagnosed between 1996 and 2011, the prevalence rates of PNI, lymphatic invasion, and vascular invasion were 29.2%, 5.2%, and 1.9%, respectively (29). Therefore, the prevalence of PNI appears markedly higher compared with both lymphatic invasion and vascular invasion. The question as to whether the Domain-KEY algorithm could be useful in distinguishing PNI from lymphatic invasion and vascular invasion on the same pathological specimen remains unanswered. Future *ad hoc* investigations should work to address this research question.

There are limitations to this study. First, our findings should be considered as hypothesis-generating data due to the small sample size. Independent validation of the Domain-KEY algorithm in large clinical cohorts, including additional analyses of variability and accuracy, will be paramount to confirm and expand our pilot findings. In this scenario, a higher number of training data is expected to improve the accuracy of the model for identifying PNI. Second, noisy signals from vascular, muscle, and lymphatic structures resulted in a decreased specificity; additional refinement steps are needed to address this issue. Third, the presence of fibroblasts and inflammatory cells surrounding adjacent nerves sporadically led to classification errors. However, inflammatory cells are generally smaller than tumor cells and fibroblasts are characterized by a typical fusiform shape with an elongated nucleus. This issue might be addressed in future studies through the use of specific size and shape filters. Finally, it can be argued that the role of immunohistochemistry for the detection of PNI remains unclear. While numerous immunohistochemical markers can be a valuable diagnostic aid in the field of OCSCC pathology, none of them has entered routine practice (30, 31). Currently, diagnostic assessment of OCSCC continues to rely on traditional histopathology.

## Data availability statement

The original contributions presented in the study are included in the article/**Supplementary Material**. Further inquiries can be directed to the corresponding author.

## Ethics statement

This study was reviewed and approved by ETHICS APPROVAL Pathology slides of OCSCC were obtained from the Tissue Bank of the Chang Gung Memorial Hospital, Linkou (Taoyuan, Taiwan). The local Institutional Review Board (identifier: 2020-00-346B) granted ethical approval to process and analyze all data. The patients/participants provided their written informed consent to participate in this study.

## Author contributions

Study concept and design: L-YL, Y-CL and C-TL. Data analysis and interpretation: All authors. Manuscript drafting or critical revision for important intellectual content: All authors. Final approval of the manuscript: All authors. All authors contributed to the article and approved the submitted version.

## Funding

This study received funding from JelloX Biotech Inc. (grant number 2020-00-346B). The funder was involved in the deep learning process and analysis. CL had full access to all of the data in this study and take complete responsibility for the integrity of the data and the accuracy of the data analysis.

## Acknowledgments

The authors wish to acknowledge the Biobank of Chang Gung Memorial Hospital, Linkou, for providing the slides used in this study. This work was financially supported by JelloX Biotech Inc. (grant number 2020-00-346B).

## Conflict of interest

Y-HH, Y-AC, Y-CL, MD-TC and Y-YL are employees of JelloX Biotech Inc.

The remaining authors declare that the research was conducted in the absence of any commercial or financial relationships that could be construed as a potential conflict of interest.

This study received funding from JelloX Biotech Inc. (grant number 2020-00-346B). The funder was involved in the deep learning process and analysis.

## Publisher’s note

All claims expressed in this article are solely those of the authors and do not necessarily represent those of their affiliated organizations, or those of the publisher, the editors and the reviewers. Any product that may be evaluated in this article, or claim that may be made by its manufacturer, is not guaranteed or endorsed by the publisher.
