# Supplementary material for: A domain knowledge enhanced yield based deep learning classifier identifies perineural invasion in oral cavity squamous cell carcinoma
Source: Front Oncol. 2022 Oct 24;12:951560. doi: 10.3389/fonc.2022.951560 (PMC9638412; doi:10.3389/fonc.2022.951560)
Supplement: Supplementary file 1 [file Table_1.pdf]

SUPPLEMENTARY TABLE 1. Confusion Matrix and Testing Performance of Different Models for Identifying Perineural Invasion (Regions of Interest): Domain-KEY AI model *versus* HRNet_v2 model

(A) CGMH method (Domain-KEY AI; number of nerve structures, n = 191)

	Actual: positive (*n*)	Actual: negative (*n*)	
Predicted: positive	TP (116)	FP (14)	
Predicted: negative	FN (7)	TN (54)	
	Sensitivity: 94.31%	Specificity: 79.41%	Accuracy: 89.01%

(B) CGMH method (invasion model only; number of nerve structures, n = 191)

	Actual: positive (*n*)	Actual: negative (*n*)	
Predicted: positive	TP (122)	FP (68)	
Predicted: negative	FN (1)	TN (0)	
	Sensitivity: 99.19%	Specificity: 0.00%	Accuracy: 63.87%
